# Widespread anatoxin-a detection in benthic cyanobacterial mats throughout a river network

**DOI:** 10.1371/journal.pone.0197669

**Published:** 2018-05-18

**Authors:** Keith Bouma-Gregson, Raphael M. Kudela, Mary E. Power

**Affiliations:** 1 Department of Integrative Biology, University of California, Berkeley, California, United States of America; 2 Ocean Sciences Department, University of California, Santa Cruz, California, United States of America; INRA, FRANCE

## Abstract

Benthic algae fuel summer food webs in many sunlit rivers, and are hotspots for primary and secondary production and biogeochemical cycling. Concerningly, riverine benthic algal assemblages can become dominated by toxic cyanobacteria, threatening water quality and public health. In the Eel River in Northern California, over a dozen dog deaths have been attributed to cyanotoxin poisonings since 2000. During the summers of 2013–2015, we documented spatial and temporal patterns of cyanotoxin concentrations in the watershed, showing widespread distribution of anatoxin-a in benthic cyanobacterial mats. Solid phase adsorption toxin tracking (SPATT) samplers were deployed weekly to record dissolved microcystin and anatoxin-a levels at 10 sites throughout the watershed, and 187 *Anabaena*-dominated or *Phormidium-*dominated cyanobacterial mat samples were collected from 27 locations to measure intracellular anatoxin-a (ATX) and microcystins (MCY). Anatoxin-a levels were higher than microcystin for both SPATT (mean MCY = 0.8 and ATX = 4.8 ng g resin^-1^ day^-1^) and cyanobacterial mat samples (mean MCY = 0.074 and ATX = 1.89 μg g^-1^ DW). Of the benthic mats sampled, 58.9% had detectable anatoxin-a (max = 70.93 μg g^-1^ DW), while 37.6% had detectable microcystins (max = 2.29 μg g^-1^ DW). SPATT cyanotoxin levels peaked in mid-summer in warm mainstem reaches of the watershed. This is one of the first documentations of widespread anatoxin-a occurrence in benthic cyanobacterial mats in a North American watershed.

## Introduction

Cyanobacteria are a diverse phylum of photosynthetic bacteria that are globally distributed across aquatic environments [1,2]. Under certain environmental conditions, their accrual rates increase, resulting in cyanobacterial blooms. Water quality can be degraded by cyanobacterial blooms [3], especially when they produce cyanotoxins, secondary metabolites that are harmful to humans and other organisms [4,5]. Though most research has focused on planktonic blooms in lakes or estuaries, benthic cyanobacterial mats in rivers can also impair water quality in fluvial systems [6]. Cyanotoxins produced within mats threaten public health for those using rivers for recreation, drinking water, or irrigation [6,7]. Furthermore, benthic cyanobacterial mats often detach from the riverbed and float downstream to accumulate in pools or along channel margins, increasing the types of habitats where cyanobacterial biomass may accrue [8,9]. Knowledge of the environmental controls of freshwater benthic cyanobacterial mats lags behind our understanding of planktonic cyanobacterial ecology [6,10–12]. Improved understanding of benthic cyanobacterial ecology is needed to identify and predict where and when cyanobacterial proliferations may negatively affect aquatic ecosystems and human health.

Benthic cyanobacterial blooms have been detected in rivers worldwide [6–8,13,14]. In New Zealand, benthic cyanobacterial blooms increased in the mid-2000s and have been documented in over 100 rivers in the country [8], and dog deaths from benthic cyanobacteria have also been reported in France [15,16]. In California, a statewide survey of benthic periphyton found potentially toxigenic cyanobacterial taxa in over 300 of 1000 samples, and the cyanotoxin microcystin (MCY) was found in 123 of 368 samples tested across the state [13]. Microcystin is a hepatotoxic cyclic-peptide that inhibits protein phosphatase activity in liver cells, causing liver hemorrhaging [17]. The Eel River in Northern California, where over a dozen dog deaths have been linked to cyanotoxin poisonings since 2000, is one the first locations of a documented anatoxin-a (ATX) dog poisoning in North America [18,19]. Anatoxin-a is a neurotoxic alkaloid produced by cyanobacteria that inhibits neuromuscular receptors by disrupting cellular ion channels, resulting in muscle failure and sometimes death [20–22]. Most cyanotoxins are intracellular [23], and so ingestion of cyanobacterial biomass is often necessary to receive a large cyanotoxin dose. However, dissolved cyanotoxins are released into the water when cyanobacterial cells lyse [23], which especially occurs when benthic mats senesce and die.

Monitoring cyanotoxins and establishing water quality regulatory guidelines in rivers are challenging [8], partly due to the diversity of toxigenic cyanobacterial taxa and cyanotoxin molecules. Additionally, toxin production by a given cyanobacterial taxon can be variable, and our understanding of environmental triggers for toxin production is rudimentary [24–27]. Solid phase adsorption toxin tracking (SPATT) samplers provide a time-integrated method of detecting cyanotoxins [28–30]. SPATT samplers are adsorptive resins placed in a water body for a given time period. Dissolved cyanotoxin molecules adsorb and accumulate onto the resin, providing a relative estimate of dissolved cyanotoxin levels in the water column over the deployment time. By sequentially deploying and retrieving SPATT samplers at a location, it is possible to generate a time series of relative toxin levels. Additionally, SPATT samplers accumulate cyanotoxins even at low environmental concentrations, so SPATT samplers often accumulate cyanotoxins at sites where water column grab samples fail to measure cyanotoxins [28,31,32]. However, variation in adsorption and equilibration kinetics of molecules onto SPATT resins due to changes in environmental conditions over the deployment period limit the quantitative interpretation of SPATT values [30]. SPATT sampling can be accompanied by other independent sampling methods (e.g. grab samples) to corroborate and help interpret the patterns in the SPATT data. SPATT samplers have been used to track the dynamics of microcystins in California’s Monterey Bay [33–35] and San Francisco Estuary [36], and micro-algal toxins in French coastal lagoons [37,38], and SPATT samplers are well-suited to monitor in rivers where benthic cyanobacteria may release transient pulses of cyanotoxins, which are then transported downstream [31].

Motivated by multiple dog deaths reported in Northern California rivers, and minimal understanding of the benthic toxic cyanobacteria responsible for them, we conducted watershed scale surveys across the Eel River with the goals of 1) describing the spatial and temporal patterns of anatoxin-a and microcystin in the watershed and 2) identifying benthic sources of cyanotoxins in the watershed. We focused on two commonly occurring toxin groups, anatoxin-a and microcystins. Anatoxin-a was chosen due to its prior detection in the watershed [18], while microcystin was chosen given its common previous detection in California watersheds [13,35,36,39]. Benthic cyanobacterial mats dominated by the genera *Anabaena* or *Phormidium* were the focus of this study due to their common occurrence as sizeable mats (several m^2^) in the watershed.

## Material and methods

### SPATT monitoring sites

**Fig 1:**
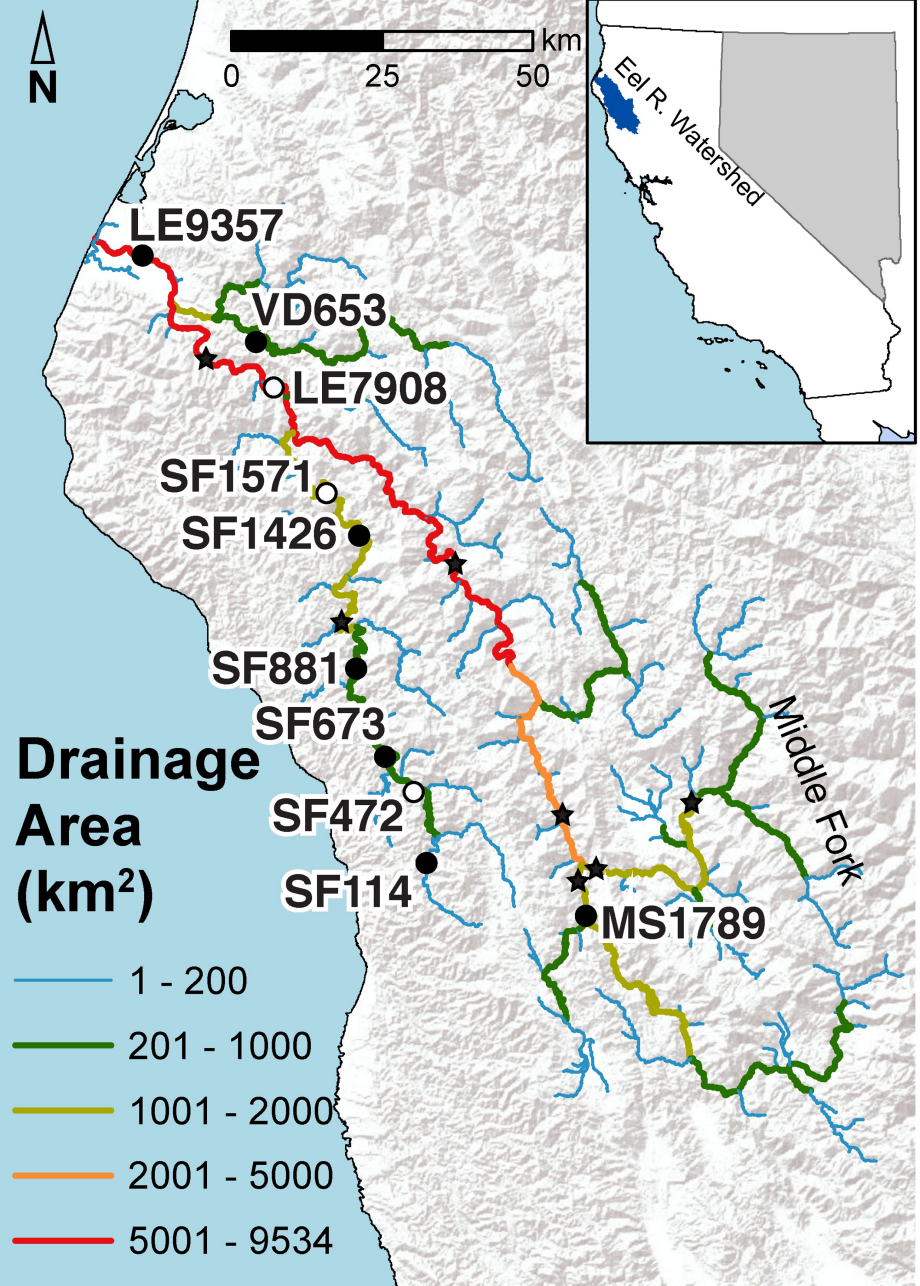
Map of cyanotoxin monitoring locations in the Eel River. Site names indicate sub-watershed (letters) and drainage area in km^2^ (numbers). Three sites with white circles were only monitored in 2014, while sites with black circles were monitored in 2013 and 2014. Stars represent sites that were monitored monthly in 2015. (SF = South Fork, MS = Mainstem, LE = Lower Eel, VD = Van Duzen).

The Eel River in Northern California drains 9547 km^2^ along the northern California Coast Range Mountains (Fig 1). In 2013 and 2014, SPATT deployment sites were established in the South Fork (SF), Lower Eel (LE), Mainstem (MS), and Van Duzen (VD) sub-watersheds (Fig 1) at sites in wadeable reaches where the water was flowing and well mixed. Sites spanned a temperature gradient with cooler water temperatures downstream near the mouth and closer to the headwaters (S1 Fig). SPATT samplers were attached to a metal pipe that was hammered into the riverbed in the thalweg. The sampler was attached to the stake at approximately half of the water depth (~30–50 cm above the bed). From mid-June until late September, every 5–10 days (S1 Table), the samplers were removed from the river and immediately replaced with a new sampler. Upon removal, samplers were rinsed with river water, and then stored in plastic bags at -20°C until toxins were extracted. In 2015, additional sites were established in the South Fork (1), Lower Eel (1), Mainstem (3), and Middle Fork (MF) Eel River (2). All samplers in 2015 were deployed for about a month (from mid-May to October) to detect the presence or absence of cyanotoxins at monitoring sites (S1 Table). Attached to the bottom of each SPATT sampler pole was a DS1922L ibutton temperature logger (Maxim Integrated, San Jose, CA, USA). Ibuttons were vacuum-sealed in a plastic bag and placed inside a 1.9 x 7.6 cm PVC pipe with holes drilled into the side. Temperature was recorded every 30 min.

### SPATT sampler construction and cyanotoxin analysis

**Fig 2:**
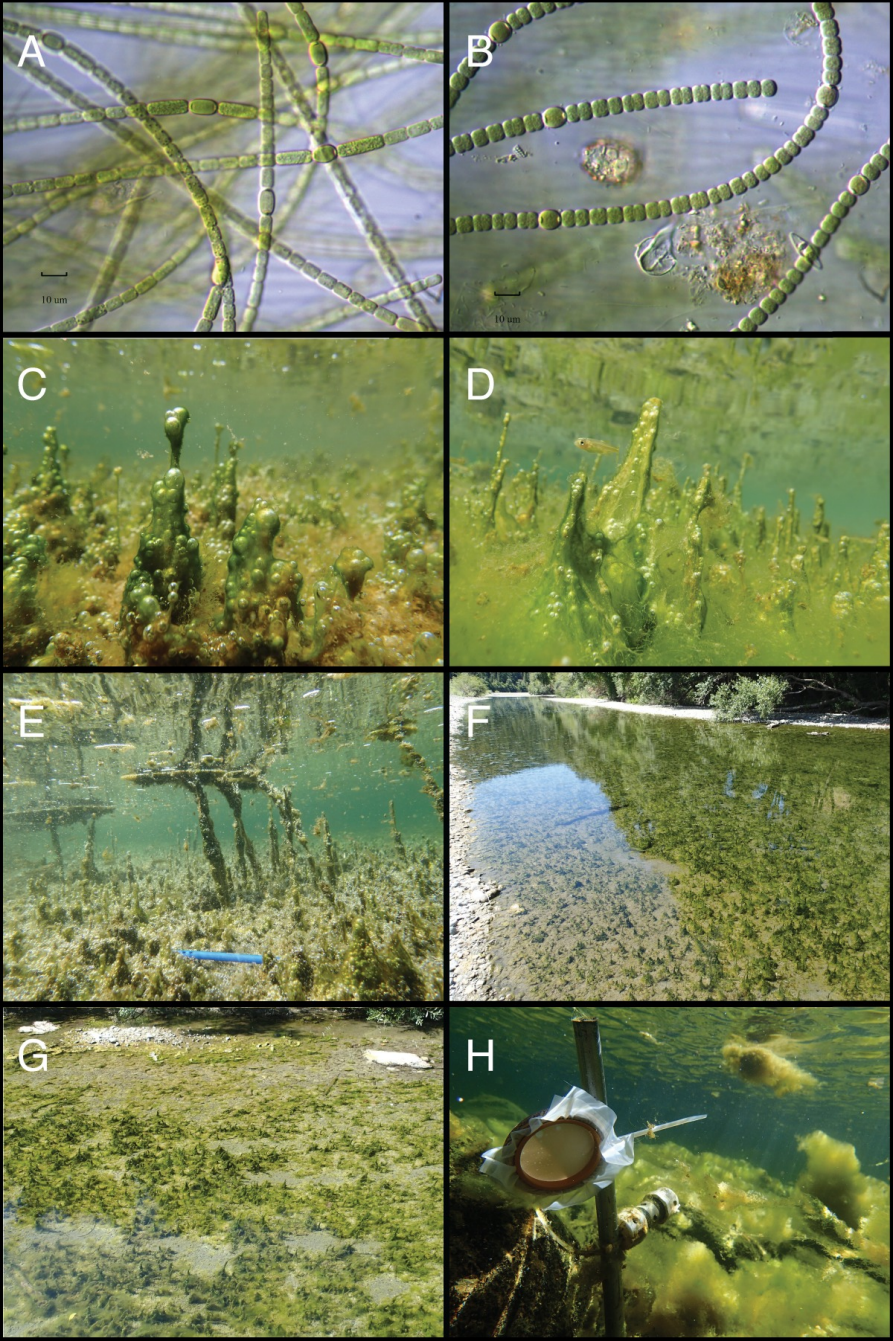
Anabaena in the Eel River. A-B) Micrographs of *Anabaena* cells (400x); C-E) Dark-green *Anabaena* “spires” growing on top of senescing macro-algae *Cladophora glomerata*; F-G) *Anabaena*-dominated mats on riverbed at bottom of shallow pools; H) SPATT sampler deployed in the Eel River.

The construction of SPATT samplers was adapted from Kudela [33] and Lane et al. [40]. To make SPATT samplers, 3 g of HP20 DIAION resin (hereafter, HP20) were sandwiched between two 10×10 cm squares of 118 μm Nitex mesh and placed in a 6.3 cm diameter embroidery hoop ring (Westex/Caron Flex Hoop rings) (Fig 2H). Immediately after construction, SPATT samplers were submerged in 100% HPLC grade methanol (Fisher A456) for ~24 hours to activate and clean the resin. Then the methanol was rinsed off by agitating the ring for 30–60 s three times in 500 mL of Milli-Q water. After rinsing, samplers were placed in plastic bags with 20 mL of Milli-Q water and stored in the dark at 4°C until deployment in the river.

Toxin extraction from SPATT followed methods in Kudela [33] and Gibble and Kudela [35]. SPATT samplers were thawed, and the resin rinsed with Milli-Q water. Then the resin was poured into a disposable liquid chromatography column and placed on a vacuum manifold. Toxins were extracted from the resin with consecutive 10, 20, 20 mL rinses of a 50% solution of methanol (Fisher A452) and Milli-Q water.

A 1 mL subsample from each of the three extracts was analyzed separately using liquid chromatography mass spectrometry (LC-MS) with select ion monitoring. The concentrations from the three SPATT extracts were then summed to give nanograms (ng) of cyanotoxin per gram resin per day deployed. Microcystin and anatoxin-a concentrations were measured on an Agilent 6130 Liquid Chromatography-Mass Spectrometry (Agilent, Santa Clara, USA) system with a Phenomenex Kinetex C18 column (microcystins; Phenomenex, Torrance, CA, USA) and Cogent Diamond-Hydride column (anatoxin-a; MicroSolv Technology Corporation, Leland, NC, USA) and direct-injection of 20 μL. The microcystin analysis followed Gibble and Kudela [35] and Mekebri [41]. The LC-MS measured four microcystin congeners –LR, –YR, –RR, and –LA; these values were then summed together. Anatoxin-a analysis followed Cogent method 141 [42]. Calibration was performed using certified reference materials (anatoxin-a: National Research Council of Canada CRM ATX and Tocris anatoxin-a fumarate; microcystin: Fluka 33578 and Sigma–Aldrich M4194) with a minimum of five calibration points for each batch of samples, with analytical blanks and matrix blanks included in each run. The detection limits of the LC-MS for anatoxin-a and microcystin were 0.25 and 0.01 parts per billion (ppb), respectively. Other anatoxin-a variants, such as homoanatoxin-a, are also produced by benthic cyanobacteria. Because certified standards do not exist for these variants [43], however, only anatoxin-a was measured in this study, though it is likely that these anatoxin-a variants were present in our samples, as these molecules have been found to often co-occur in New Zealand rivers [8,43].

The detection limit of dissolved cyanotoxins in the river by SPATT resins and the calibration of SPATT values to dissolved cyanotoxin concentrations were not investigated. These relationships are challenging to quantify, since SPATT adsorption is a function of environmental conditions such as hydraulics, water chemistry, temperature, light, and microbial activity. SPATT is therefore considered a semi-quantitative method [30].

### SPATT anatoxin-a adsorption and extraction

The adsorption and extraction efficiencies of HP20 resin and microcystins are described in Kudela [33], and the method is estimated to recover ~60–100% of microcystins from the resin. Since Kudela [33] only studied microcystin, we conducted three laboratory experiments to characterize the adsorption and extraction dynamics for anatoxin-a and HP20 resin. First, to understand the degradation dynamics of the anatoxin-a molecule, anatoxin-a standards were added to a single 125 mL flask of 0.2 μm filtered Eel River water and single 125 mL flask of Milli-Q water. Both flasks were constantly agitated (70 rpm) at room temperature and illuminated at 125 μmol m^-2^ s^-1^. These flasks were sampled over 48 hours to track changes in anatoxin-a concentrations due to degradation or interactions with other dissolved molecules, which limit detection via LC-MS. Second, to assess the adsorption and extraction dynamics of the HP20 resin, anatoxin-a standards were added to triplicate 0.2 μm filtered Eel River and Pinto Lake water in 125 mL Erlenmeyer flasks with constant agitation. Pinto Lake is a 44 ha lake in Santa Cruz County that experiences frequent *Microcystis* blooms. It was chosen as a second environmental water source due to its proximity to laboratory facilities. Then, 3 g of HP20 resin in a SPATT ring was added to each flask and the flasks regularly sampled to measure anatoxin-a concentrations. Filtered Eel or Pinto water without a SPATT ring were used as a control. To estimate recovery efficiency of anatoxin-a from the SPATT resin, anatoxin-a was extracted from the HP20 resin used in the second experiment with sequential volumes of 50% methanol, as described above. The recovery efficiency is calculated by dividing the total anatoxin-a extracted off the SPATT by the total anatoxin-a at the beginning of the experiment, with the control (no SPATT) samples used to account for variability due to sample handling and analysis.

### Cyanobacterial mat collections

**Fig 3:**
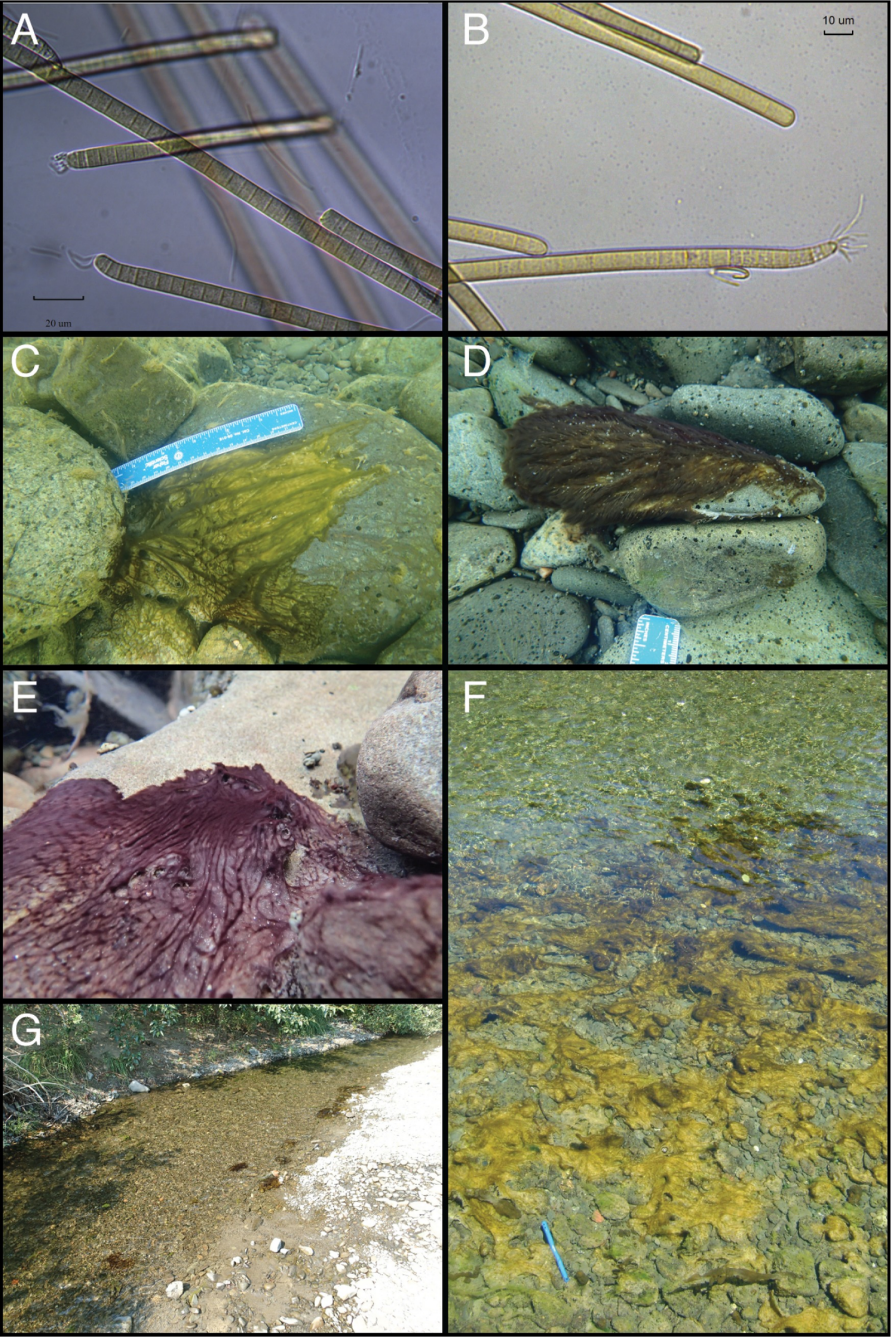
Phormidium in the Eel River. A-B) Micrograph of *Phormidium* cells (400x); C-E) Underwater photographs of *Phormidium* growing on cobbles; F-G) Looking down on brown or orange patches of *Phormidium* mats in the river (blue thermometer is 15 cm long).

From June through August in 2014–2015, mats dominated by *Anabaena* or *Phormidium* (Figs 2 and 3) were sampled opportunistically on different survey trips around the watershed, or when mats were present near SPATT monitoring sites (S2 Table). All samples (including SPATT monitoring sites) were collected either at sites with public unrestricted access to the river channels, on private land with permission granted through coordination with landowners and the non-profit Eel River Recovery Project (www.eelriverrecovery.org), or at the Angelo Coast Range Reserve (angelo.berkeley.edu) with permission from the reserve manager. Mats were macroscopically identified by color and morphology, and then sub-samples collected to confirm taxonomic identification with microscopic identification of these two genera at 400x (Komarek 2013) on a Nikon Optiphot 2 microscope (Nikon Instruments, New York, USA). Other Cyanobacterial genera, diatoms (Bacillariophyceae), and green algae (Chlorophyta) were present in the mats, but the macroscopic morphology of the mats is formed by the *Anabaena* or *Phormidium*, so we refer to the mats as *Anabaena*-dominated or *Phormidium*-dominated. The genus *Anabaena* was recently split with all planktonic species containing gas vesicles moved to *Dolichospermum*, while benthic species (without gas vesicles) remain in *Anabaena* [44,45].

Mats were sampled with a 3.8 cm diameter PVC pipe delimiter. For a given cyanobacterial mat, 3–5 sub-samples were collected and combined into a single composite (median = 0.5 g DW, S2 Table) sample from the mat, for a total of 187 samples from 27 different places in the watershed (S2 Table). In the lab, after macroinvertebrates were removed from the sample, 50–150 mL of water was added to the mat sample, which was then homogenized in a blender, and the volume of the homogenate recorded. A 10–15 mL subsample was collected for cyanotoxin analysis, placed in a 20 mL glass scintillation vial, and frozen at -20°C until toxin analysis. An additional 10–15 mL subsample of the homogenized mat was collected and dried at 50°C for 24–48 hours and then weighed to the nearest 0.1 mg. Toxin concentrations from mat samples were standardized by the dry weight of the additional sub-sample and expressed as micrograms (μg) of toxin molecule per gram of mat dry weight (DW).

For intracellular cyanobacterial mat toxin analysis, samples were thawed and 3 mL of sample added to a glass culture tube. Then, 3 mL of 100% methanol were added and the tube was sonicated for 30 s using a probe sonicator at ~10W power (Fisher Sonic Dismembrator 100; Thermo Fisher Scientific, Massachusetts, USA) then centrifuged for 5 min. at 1083 rcf [46]. For anatoxin-a analysis, a 1 mL subsample of the supernatant was taken and 0.2 μm filtered into an LC-MS vial [43]. Microcystin subsamples were cleaned [41] using solid phase extraction (SPE) with a Baker C18 column by subsampling 3 mL of the supernatant and adding 35 mL of acid water (0.1% formic acid and 0.05% trifluoroacetic acid). This mixture was then added to the SPE column and eluted off the column with 2 mL of 90% methanol acidified with 0.1% trifluoroacetic acid. Then 1 mL of eluate was added to a LC-MS vial. The anatoxin-a and microcystin concentrations in the vials were measured using LC-MS with the same method described for SPATT samples.

### Cyanotoxin water samples

To test for total cyanotoxins in the water column, in 2015, 15 mL unfiltered water samples were collected in glass scintillation vials from ~15 cm below the water surface at sites throughout the watershed, placed in a cooler until returned to the laboratory, and then frozen at -20°C until analysis. Samples were thawed and a 3 mL subsample taken and combined with 3 mL of 100% HPLC grade methanol (Fisher A542), sonicated for 30 s, centrifuged at 1083 rcf for 5 min, and then 0.2 μm filtered into a LC-MS vial [33]. Samples were tested for microcystins and anatoxin-a using LC-MS with the same method described for SPATT samples. In June and July 2016, 26 river water samples from the South Fork Eel River were collected from 20–30 cm below the river surface and a 300–500 mL random sub-sample filtered onto 25 mm diameter 0.45μm mixed cellulose ester filters (Millipore HAWP02500; MilliporeSigma, MA, USA). The filter was clarified with immersion oil, and then *Anabaena* cells counted on the entire filter at 400x (Nikon Optiphot 2) to estimate the number of *Anabaena* cells per milliliter of river water.

### Statistics

Generalized linear binomial models were used to test for differences in the presence and absence of anatoxin-a or microcystins in *Anabaena*-dominated and *Phormidium*-dominated mats after intracellular cyanotoxin concentrations were transformed into presence/absence values. To test differences in cyanotoxin concentrations between *Anabaena-*dominated and *Phormidium*-dominated mats, all concentrations below the detection limit were removed from the data and remaining values were log_10_ transformed to assume normality. Then, a Welch’s t-test was applied to the data. Alpha was set at 0.05, and all p values were Bonferroni corrected to account for multiple comparisons. Statistical analyses were performed with R v.3.3.2 [47] using the lme4 package [48].

## Results

### SPATT anatoxin-a adsorption and extraction

In laboratory experiments, anatoxin-a concentrations remained relatively stable over 48 hours in Milli-Q water, but decreased in filtered Eel River water (S2A Fig). When SPATT resins were placed in flasks, most anatoxin-a adsorbed onto the SPATT within 12 hours, thereafter the adsorption rate decreased (S2B Fig). The changes in anatoxin-a concentrations over time were similar between the Eel River and Pinto Lake water samples. The anatoxin-a concentrations in the control flasks remained stable, with minimal decreases in anatoxin-a over 72 hours. However, between 72 and 78 hours the anatoxin-a degradation rate increased for all samples. Anatoxin-a recovery from SPATT resin was 58 ± 2.0% for Eel River water and 73 ± 2.4% (mean ± 1 SD) for Pinto Lake water of the total amount of anatoxin-a present at the start of the experiment.

### SPATT monitoring

**Fig 4:**
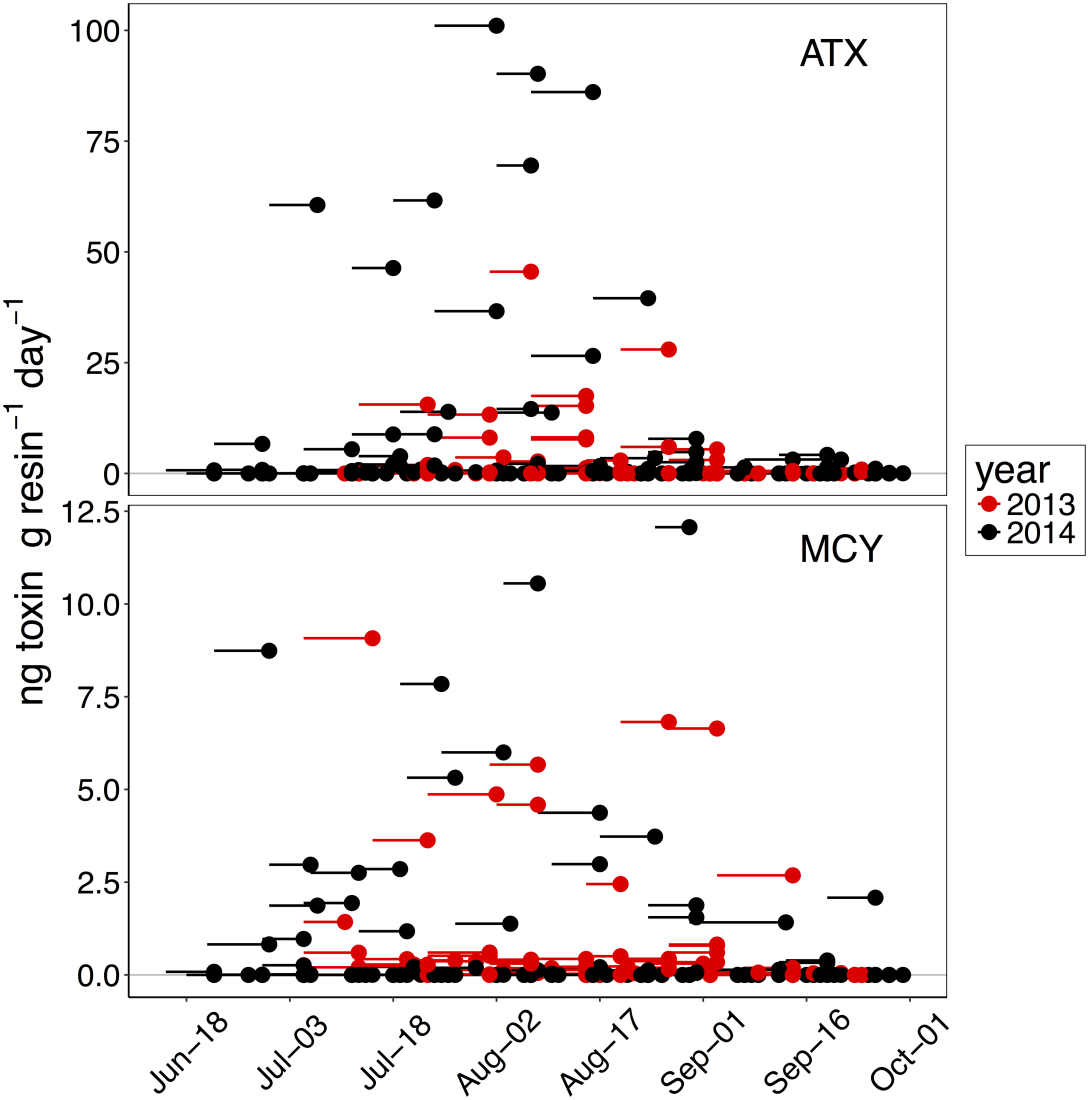
Anatoxin-a (ATX) and microcystin (MCY) accumulations on SPATT samplers in the Eel River for 2013 and 2014. Each point represents the retrieval date of a single sampler, and the line extends back to the day the sampler was deployed. Note the different scales of the y-axis for anatoxin-a and microcystin.

SPATT samplers accumulated more anatoxin-a (mean = 4.8, max = 101.1 ng ATX g resin^-1^ day^-1^) than microcystin (mean = 0.8, max = 12.1 ng MCY g resin^-1^ day^-1^) at our monitoring sites in the Eel River (Fig 4), but accumulated microcystins more frequently than anatoxin-a in 2013, but not 2014 (Table 1). Most of the microcystin SPATT values were below 0.001 ng MCY g resin^-1^ day^-1^, with only the SF114 SPATT sampler having a median value >0.5 ng MCY g resin^-1^ day^-1^ (Figs 4 and S3). The microcystin levels were primarily comprised of the congener–LR, which consistently accumulated at higher amounts than the other three congeners (S4 Fig).

**Table 1:** The percentage of SPATT samplers detecting anatoxin-a (ATX) and microcystin (MCY).

ID	2013 ATX	2014 ATX	2013 MCY	2014 MCY
LE9357	27% (3/11)	21% (3/14)	73% (8/11)	21% (3/14)
LE7908	NA	50% (5/10)	NA	40% (4/10)
SF1571	NA	38% (5/13)	NA	21% (3/14)
SF1426	100% (9/9)	67% (8/12)	78% (7/9)	33% (4/12)
SF881	89% (8/9)	77% (10/13)	44% (4/9)	38% (5/13)
SF673	88% (7/8)	92% (11/12)	75% (6/8)	33% (4/12)
SF472	NA	43% (3/7)	NA	29% (2/7)
SF114	33% (3/9)	62% (8/13)	100% (9/9)	100% (13/13)
VD653	45% (5/11)	47% (8/15)	73% (8/11)	60% (9/15)
MS1789	0% (0/9)	45% (5/11)	89% (8/9)	20% (2/10)
Overall	53% (35/66)	54% (65/120)	76% (50/66)	41% (49/120)

Parentheses show number of samplers testing positive over total number of samplers deployed at a given site. Sites not established in 2013 are represented with NA. ID labels indicates sub-watershed and watershed area upstream of site in km^2^: LE = Lower Eel, SF = South Fork, VD = Van Duzen, MS = Mainstem.

**Fig 5:**
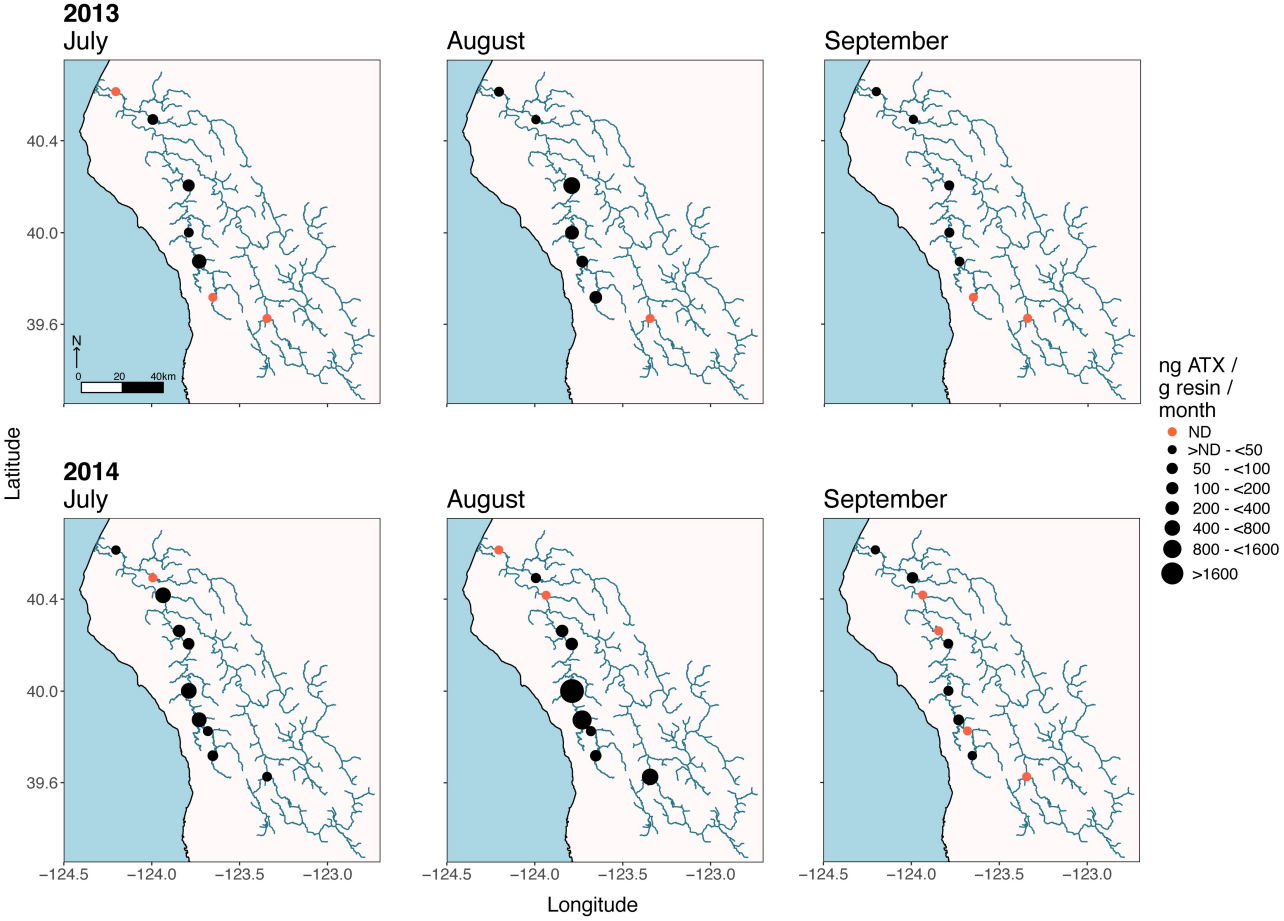
Anatoxin-a (ATX) accumulated each month from SPATT monitoring locations in 2013 and 2014. Red points indicate sites where cyanotoxins were no accumulated anatoxin-a was detected (ND) on SPATT samplers. The diameter of the black points categorize the range of cyanotoxins accumulated on the SPATT samplers for a given month.

**Fig 6:**
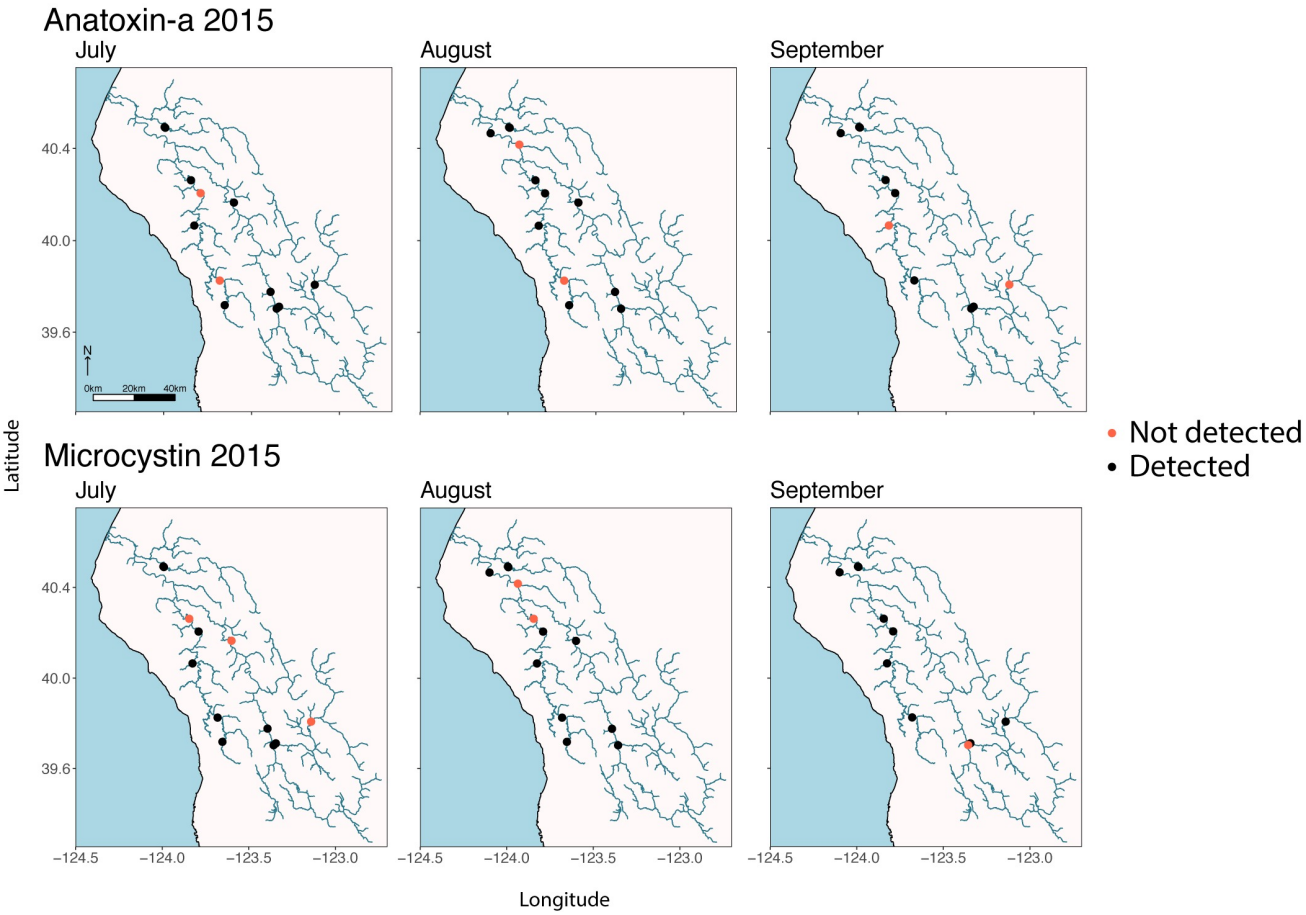
Detection of anatoxin-a and microcystin accumulated on SPATT samplers deployed in 2015 in locations throughout the Eel River watershed.

The seasonal timing of the rise and fall of SPATT-sampled anatoxin-a and microcystin levels was similar in 2013 and 2014 (Figs 4 and 5). In both years, SPATT levels start to increase in late July and remain elevated into early August, though for microcystins the increase begins earlier and elevated accumulations persist into September. Higher SPATT anatoxin-a levels were reached earlier in the season in 2014 than in 2013. By late August in both 2013 and 2014, SPATT anatoxin-a levels at all sites were close to zero and remained low until the end of September when monitoring ceased. In both 2013 and 2014, sites from middle reaches of the South Fork Eel (SF1426, SF881, and SF673) accumulated more anatoxin-a than site SF114 or sites LE9357 and LE7908, both near the mouth of the river (Fig 5). High levels of anatoxin-a accumulated in a single, early-season SPATT sample from LE7908, but subsequent samples at this location accumulated much less anatoxin-a (S3 Fig). The site on the Mainstem (MS1789) and Van Duzen (VD653) also, accumulated fewer cyanotoxins than South Fork sites. SPATT samplers in 2015 accumulated microcystin and anatoxin-a in July, August, and September at almost all sites (Fig 6), including the monitoring sites in the less populated eastern parts of the watershed.

### Cyanobacterial mat and water sampling

**Fig 7:**
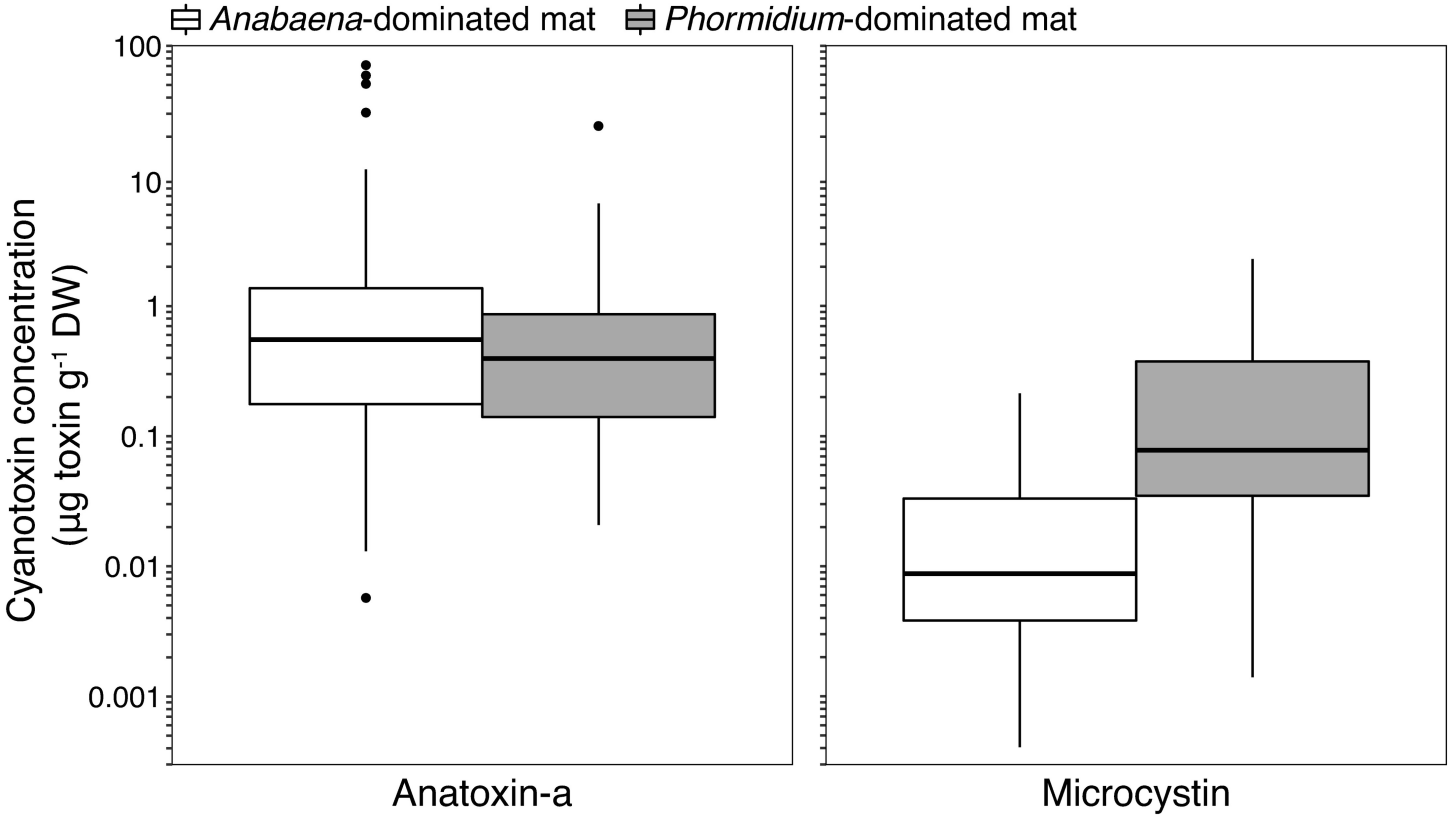
Boxplots of intracellular anatoxin-a and microcystin concentrations from cyanobacterial mats dominated by *Anabaena* (n = 116) or *Phormidium* (n = 62) collected in 2014 and 2015. Only samples with cyanotoxin concentrations above the detection limit are included in the boxplots. For anatoxin-a, there were 69 *Anabaena*-dominated and 36 *Phormidium*-dominated samples above the detection limit, and for microcystin there were 31 *Anabaena*-dominated and 36 *Phormidium*-dominated samples above the detection limit. The boxplots are plotted on a log_10_ scale, and the box represents the 25th percentile, median, and 75th percentile of the data, the whiskers extend up to 1.5x the interquartile range, and points beyond the whiskers are plotted individually.

**Fig 8:**
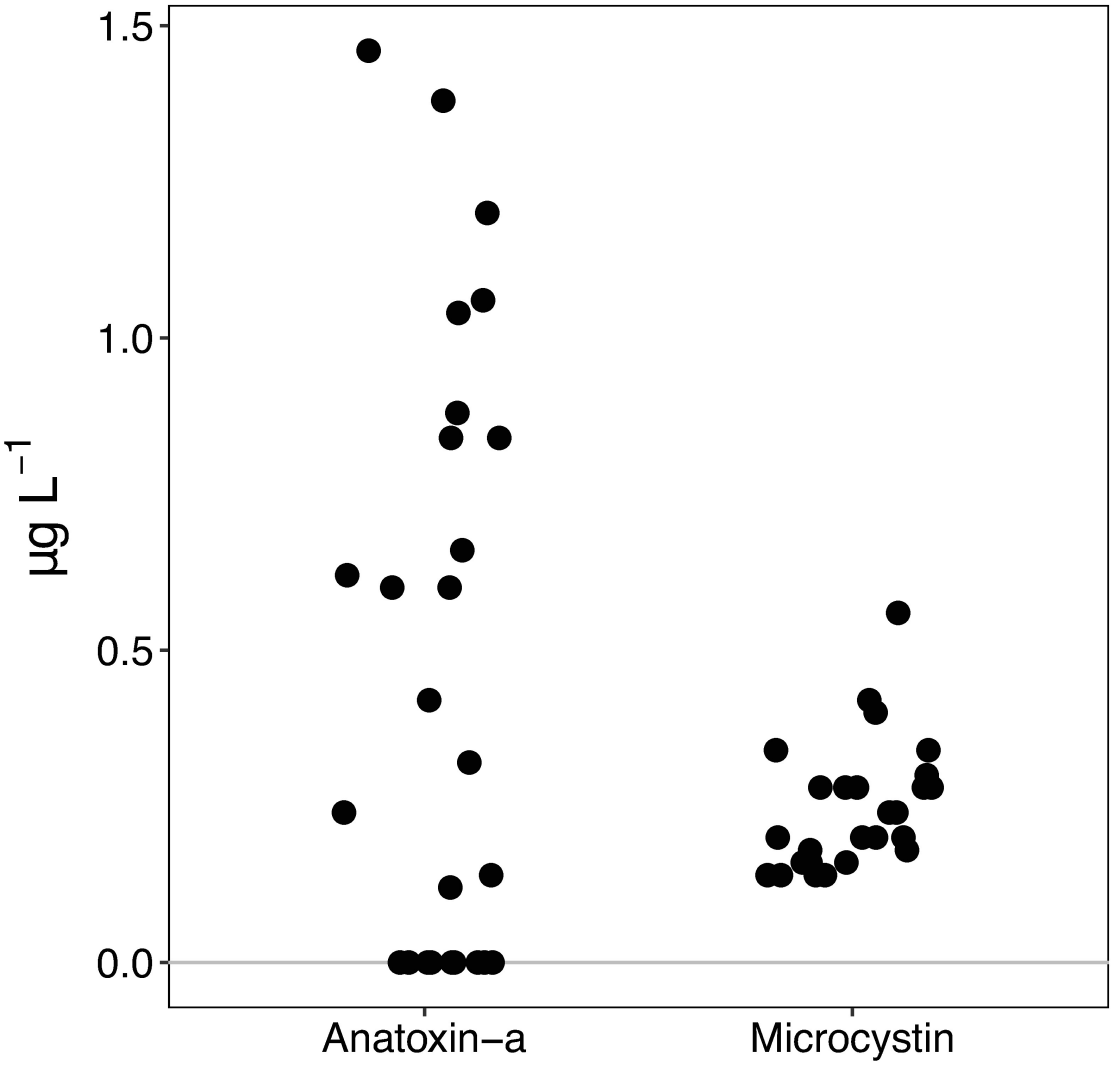
Total anatoxin-a and microcystin concentrations (dissolved plus lysed suspended cells) in unfiltered water samples collected in summer 2015 in the Eel River.

Both *Anabaena-*dominated and *Phormidium*-dominated mats (Figs 2 and 3) sampled in 2014 and 2015 contained anatoxin-a (58.9% of samples, mean = 1.89 μg g^-1^ DW) or microcystin (38.6% of samples; mean = 0.074 μg g^-1^ DW). Cyanotoxins were detected in 69.8% of *Anabaen*a and 82.3% of *Phormidium*-dominated mat samples (Table 2). Intracellular anatoxin-a was detected more frequently (p <0.01) than microcystin and at higher concentrations (Fig 7) than microcystin in the cyanobacterial mat samples (p <0.01). Anatoxin-a concentrations were not higher (p >0.05) or more frequently detected (p >0.05) in *Anabaena* samples (mean = 2.47, max = 70.93 μg ATX g^-1^ DW) compared to *Phormidium* samples (mean = 0.87, max = 24.19 μg ATX g^-1^ DW). In contrast, microcystin concentrations were higher (p <0.01) and more frequently detected (p <0.01) in *Phormidium* samples (mean = 0.20, max = 2.30 μg MCY g^-1^ DW) compared to *Anabaena* samples (mean = 0.07, max = 0.21 μg MCY g^-1^ DW) (Fig 7), with the microcystin congeners–YR and–LR having the highest concentrations across all samples (S5 Fig). Toxic mat samples were distributed across the different sub-watersheds in the Eel River catchment and toxin detection was not localized to a particular region in the catchment (S6 Fig, S2 Table). Both anatoxin-a and microcystin were measured in water samples from 2015 (Fig 8 and S3 Table), but at concentrations less than 1.5 ATX and 0.75 MCY μg L^-1^, below state recreational warning levels of 20 and 6 μg L^-1^, respectively [49]. Suspended *Anabaena* cells in the water column measured in 2016 were all at concentrations <500 *Anabaena* cells mL^-1^ (S4 Table).

**Table 2:** Percentage of *Anabaena*-dominated and *Phormidium*-dominated cyanobacterial mat samples in which microcystin (MCY) and anatoxin-a (ATX) were detected. (n = 116 *Anabaena* and n = 62 *Phormidium* samples).

Toxin detected	*Anabaena*	*Phormidium*
Both ATX & MCY	16.4%	33.9%
MCY only	10.3%	24.2%
ATX only	43.1%	24.2%
No detections	30.2%	17.7%

## Discussion

Our results document the widespread distribution of anatoxin-a in the Eel River watershed, both dissolved in the water column and within benthic cyanobacterial mats, corroborating Puschner et al. [18], which linked dog deaths in the watershed to anatoxin-a poisoning. Anatoxin-a in the Eel River watershed was consistently detected in both SPATT samples and cyanobacterial mat samples, and both sampling methods showed higher levels of anatoxin-a than microcystin. Our results likely underestimate the total concentration of anatoxin-a and its variants, since we did not measure homoanatoxin-a or di-hydro derivatives, and in New Zealand, the concentration of these variants is often higher than anatoxin-a [8]. Both *Anabaena*-dominated and *Phormidium*-dominated benthic cyanobacterial mats contained anatoxin-a, and so both can be considered potential sources of anatoxin-a. Planktonic cyanobacterial blooms have not been observed in the Eel River, and filtered water samples from the Eel River collected downstream of cyanobacterial mats in 2016 had <500 *Anabaena* cells mL^-1^ (S4 Table), much lower than “bloom” concentrations of >20000 cells mL^-1^ [50], suggesting that most biomass of toxigenic cyanobacteria is benthic.

Cyanobacterial mat samples collected *in situ* from the river were mixed microbial assemblages with macroscopically visible accruals of cyanobacteria, but multiple strains or taxa within the mats may be producing cyanotoxins. Different species or strains of both *Phormidium* [6,43,51] and *Anabaena* [6,52] produce either microcystins or anatoxin-a, and toxin concentrations in mats can even vary at the centimeter scale [43]. Due to their common occurrence and high frequency of anatoxin-a detection, we hypothesize that organisms in benthic *Anabaena*-dominated mats are the principal producers of anatoxin-a in the watershed. However, culturing of individual strains to test for toxin production and molecular analysis of toxin producing genes in cyanobacterial strains is necessary to confirm, which cyanobacteria are producing anatoxin-a and microcystin in the watershed, and how the abundance of toxin and non-toxin producing strains contributes to mat and water column concentrations [53]. Fortunately, *Anabaena*-dominated and *Phormidium*-dominated mats are morphologically distinct from other algal assemblages, resulting in easy to assign sampling categories applicable to situations, such as public health monitoring, where the types of cyanobacterial assemblages containing cyanotoxins may be more relevant than identifying the organisms within an assemblage that are producing cyanotoxins.

### SPATT anatoxin-a adsorption and extraction

Anatoxin-a adsorbed more slowly and extracted less efficiently with HP20 resin than microcystin molecules sampled and extracted by Kudela [33]. In testing different SPATT substrates for anatoxin-a adsorption dynamics, Wood et al. [31] found that HP20 adsorbed about 60% of the anatoxin-a in experimental beakers. In laboratory trials, Kudela [33] found HP20 adsorbed >90% of microcystin. Since HP20 accumulates anatoxin-a from the water column less efficiently than microcystins, SPATT data are likely to underestimate *in situ* dissolved anatoxin-a concentrations compared to microcystin concentrations. An advantage of HP20 for monitoring programs is that multiple toxins can be extracted off of a single SPATT sampler. Understanding the different adsorption and extraction dynamics of various cyanotoxins collected from the same SPATT sampler is important for interpreting and comparing the extracted cyanotoxin concentrations [29]. In high light and pH environments, extra-cellular anatoxin-a degrades within several hours [54–56]. Microbes can also accelerate the degradation of anatoxin-a [57]. Control flasks in our experiment maintained stable anatoxin-a concentrations for 48 hours, suggesting increased stability under lower light and pH [56]. Presumably, the longer a SPATT sampler is in the river, the more the degradation of older anatoxin-a molecules on the resin occurs, additionally the resin may become saturated with anatoxin-a or other compounds. More research into the stability of anatoxin-a molecules bound to SPATT resin in different light and pH environments could help optimize duration of SPATT sampler deployment [30].

### Cyanotoxin patterns in the watershed

The most upstream SPATT sampler (SF114) was the only sampler to consistently accumulate microcystins, though at much lower levels than anatoxin-a (S3 Fig). Cyanobacterial mat samples from this site did not show elevated microcystin concentrations (S2 Table (site = AN), mean = 0.015, median = <DL, μg MCY g^-1^ DW), so it is unknown what taxa are producing these microcystin molecules. *Nostoc* spp. is common near this site and some species are known to produce microcystins [7,58–60]. But *Nostoc* spp. are also found near downstream sites. In 2014, 9 *Nostoc* spp. samples were collected for cyanotoxin analysis, but none of the samples contained detectable microcystins (S2 Table). More *Nostoc* samples should be collected to determine whether they are sources of microcystins near the South Fork Eel headwaters.

Cyanotoxins rarely accumulated on the two most downstream SPATT samplers in the Lower Eel (LE9357 and LE7908). Cyanobacterial mats were also less frequently found during sampling trips in these regions. It is possible that higher downstream discharge dilutes cyanotoxin concentrations resulting in lower accumulations on SPATT samplers. During June—September in 2013 and 2014, river discharge between the two Lower Eel SPATT sites (USGS gage Scotia 11477000) was about 3.5x greater than along the South Fork between sites SF1426 and SF881 (USGS gage Miranda 114765000). However, SPATT values in the South Fork Eel were orders of magnitude higher than at Lower Eel sites, suggesting that reduced cyanotoxin production, rather than dilution, explains the low SPATT accumulations in the Lower Eel. Additionally, direct and indirect effects on cyanobacterial growth of different environmental conditions [61] in the Lower Eel compared to upstream reaches, such as smaller (less stable) bed sediments, lower temperatures (S1 Fig), and lower irradiance under the maritime coastal fog influence near the mouth, might also explain the decrease in cyanobacterial mats and cyanotoxins in the Lower Eel [62,63].

Detection distances of upstream sources of cyanotoxins for a SPATT sampler are also unknown. The SPATT sampler sites in the South Fork Eel in 2013–2014 were tens of river kilometers apart, and we found no spatial or temporal autocorrelation among the samplers. This independence suggests that samplers were not accumulating cyanotoxins produced at the same location, and could constrain the upper limits of downstream travel distance for the cyanotoxins.

Higher SPATT accumulations of anatoxin-a occurred in warmer reaches of the South Fork Eel (SF673-SF1426). However, the single Mainstem site in 2013 and 2014 (MS1789) was the hottest site (S1 Fig), but had only 5 out of 20 SPATT accumulate anatoxin-a, of which only one was greater than 1 ng ATX g resin^-1^ day^-1^, and cyanobacterial mats were never seen near this site during sampling trips 2013 and 2014. Therefore, other environmental factors besides temperature are also likely controlling cyanotoxin production in the watershed. Nitrogen to phosphorus ratios increase in the Eel River as summer progresses [64], and in New Zealand and Florida, cyanobacteria proliferate at low phosphorus concentrations [8,65]. Lower phosphorus concentrations in summer might be driving the increase in cyanobacterial biomass, but decreasing phosphorus is also correlated with increasing temperatures and decreasing river flow over time, so experiments will be needed to tease apart the independent and interactive effects of these variables on benthic cyanobacterial growth. Additionally, the relationship of dissolved nutrients to benthic algal productivity is often obscure, because benthic proliferations can rapidly deplete and store nutrients from the water column and continue to derive nutrients from local recycling or the substrate [66,67].

### Conclusion

To our knowledge, this is the first report of widespread anatoxin-a detection in an North American river. Increasingly benthic cyanobacterial mats are found to be sources of cyanotoxins in watersheds, especially streams in Mediterranean climates [6,13,68]. In addition to effects on human health from benthic cyanobacteria, benthic algal production and biomass supports aquatic and riparian food webs [66,69,70], so a shift from benthic diatoms and other algae to cyanobacterial dominance may affect grazer behavior which could alter benthic nutrient cycling, energy flow, and ecological interactions in fluvial ecosystems [66,71–73].

Monitoring methods and regulatory guidelines established in lakes and estuaries are not easily applied in rivers, where cyanobacteria are attached and aggregated into mats and river flow rapidly transports dissolved cyanotoxins away from their source. Monitoring programs must be able to address a variety of habitats where cyanobacteria could be producing cyanotoxins. Passive cyanotoxin sampling, such as SPATT, can be used in rivers to estimate cyanotoxin patterns across a watershed, and can be coupled with additional sampling methods, such as water column grab samples, benthic mat sampling, or in percent cover estimation, to monitor benthic cyanobacterial dynamics. With many different toxigenic cyanobacterial strains producing a variety of cyanotoxins, documenting the common cyanotoxins and their sources in a watershed is one of the first steps to developing efficient and effective monitoring methods and experimental designs to understand and predict the spatial distribution and effect of toxic benthic cyanobacteria on human and ecosystem health in river networks.

## Supporting information

S1 Fig Boxplots of daily mean temperatures at SPATT sites from 3-July to 31-August in 2013 and 2014. (TIF)

S2 Fig Anatoxin-a stability and adsorption efficiency. A) Anatoxin-a (ATX) stability in Milli-Q (white squares) and 0.2 μm filtered Eel River water (black circles). B) Anatoxin-a adsorption by SPATT resin in 125 mL Erlenmeyer flasks filled with 0.2 μm filtered Eel River or Pinto Lake water. No SPATT resin was placed in control flasks (white squares).
(TIF)

S3 Fig Time series of anatoxin-a and microcystin concentrations from SPATT samplers in 2013 and 2014. Sites are ordered top to bottom by watershed size. Each horizontal line represents an individual SPATT sampler and the length of the line corresponds to the number of days of deployment. LE7908, SF1571, and SF472 were only established in 2014.
(TIF)

S4 Fig Boxplots of the four different microcystin (MCY) congeners measured from SPATT samplers in 2013 and 2014. (TIF)

S5 Fig Microcystin (MCY) congeners from cyanobacterial mat samples collected in 2014 and 2015. (TIF)

S6 Fig Anatoxin-a (ATX) and microcystin (MCY) concentrations in cyanobacterial mats dominated by *Anabaena* or *Phormidium*. Samples collected in summer 2015 from Lower Eel (LE), Middle Fork (MF), Mainstem (MS), North Fork (NF), and South Fork (SF) Eel. (N = 21, 19 *Anabaena* samples and 2 *Phormidium* samples).
(TIF)

S1 Table Solid phase adsorption tracking (SPATT) data from 2013–2015 in the Eel River. (XLSX)

S2 Table Cyanobacterial mat samples collected for microcystin and anatoxin-a analyses in Eel River watershed in 2014 and 2015. (XLSX)

S3 Table Anatoxin-a and microcystin concentrations in unfiltered water samples collected in the Eel River watershed. (XLSX)

S4 Table Cell counts of *Anabaena* cells from filtered Eel River water samples. (XLSX)
