# Supplementary figures and images for: Widespread anatoxin-a detection in benthic cyanobacterial mats throughout a river network
Source: PLoS One. 2018 May 18;13(5):e0197669. doi: 10.1371/journal.pone.0197669 (PMC5959195; doi:10.1371/journal.pone.0197669)

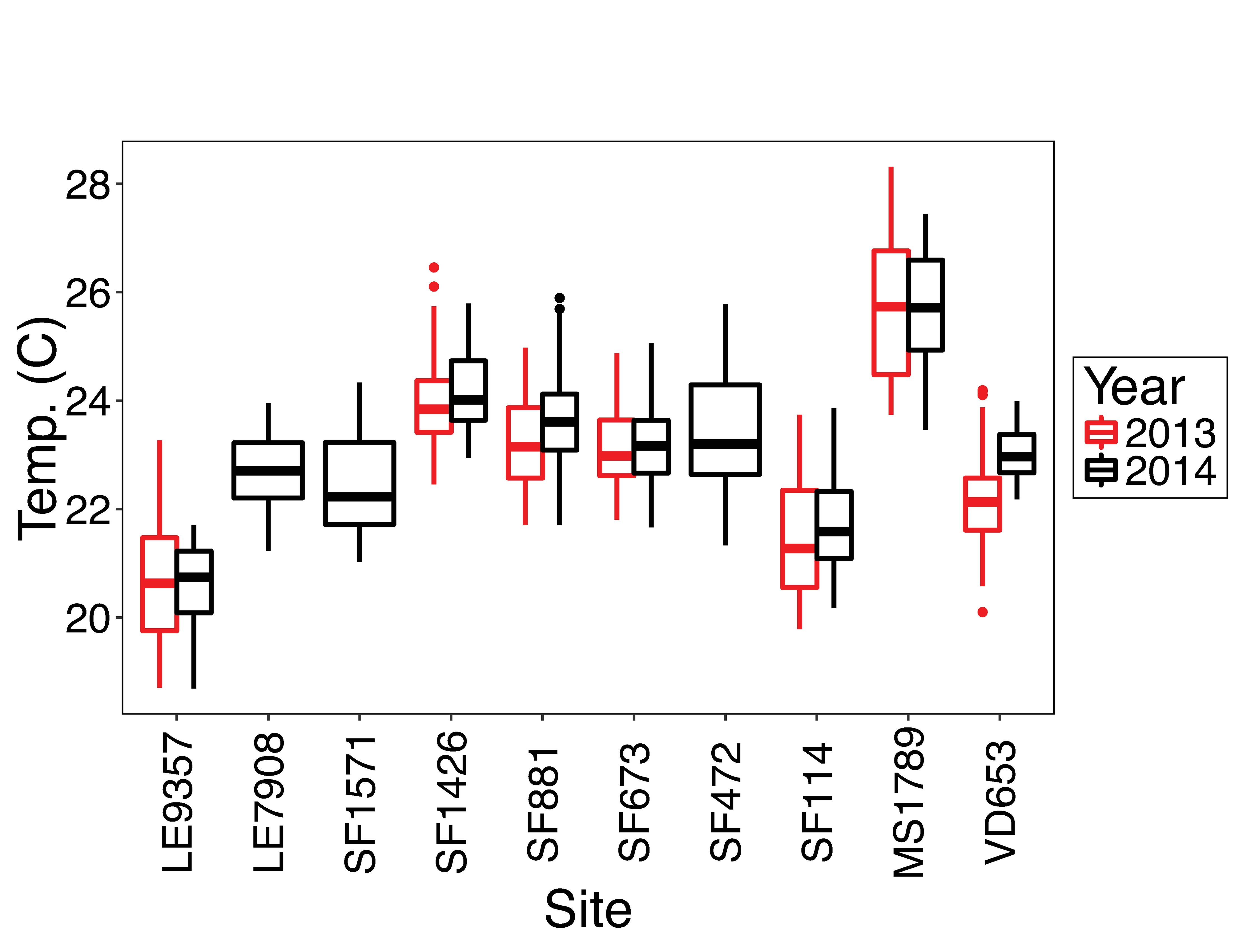

Supplement: S1 Fig — (TIF) [file pone.0197669.s001.tif]

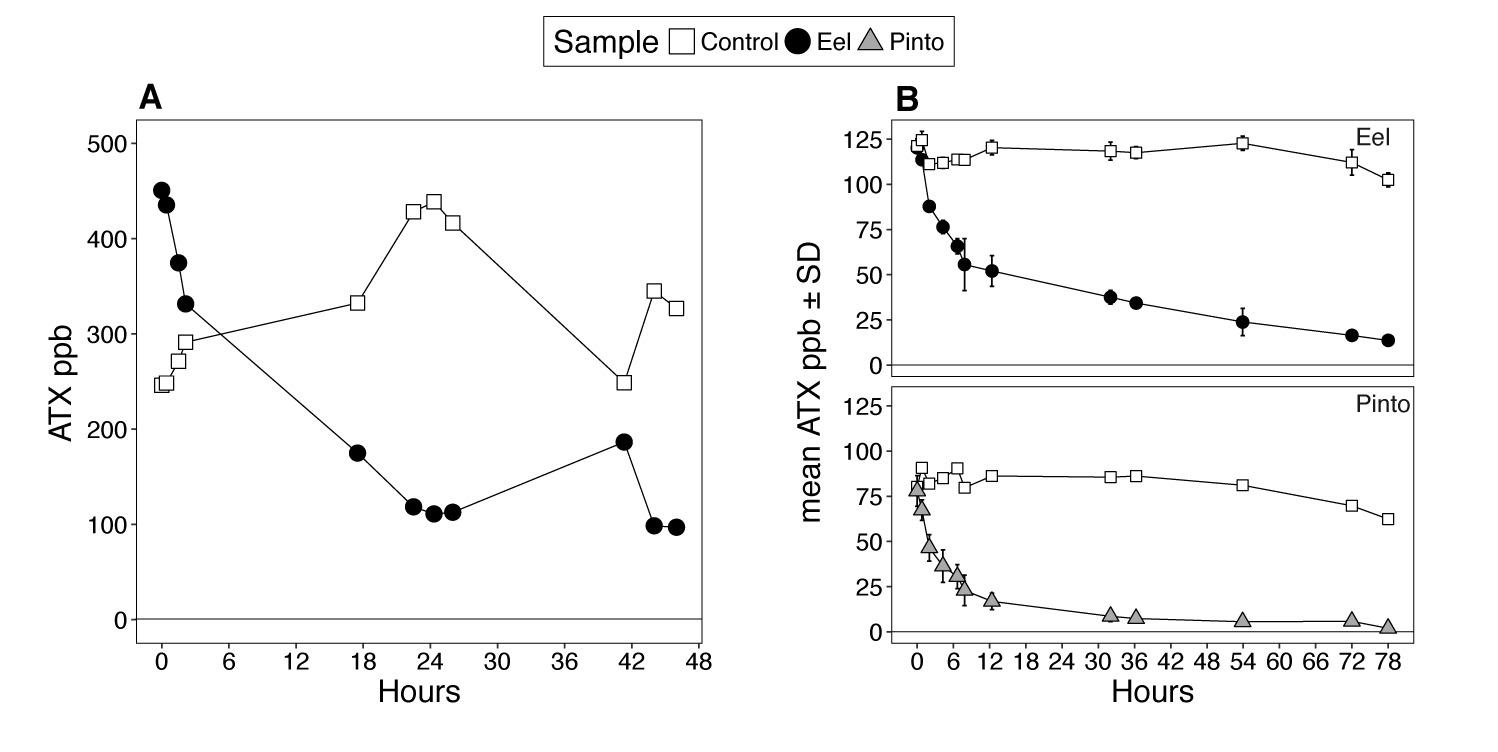

Supplement: S2 Fig — A) Anatoxin-a (ATX) stability in Milli-Q (white squares) and 0.2 μm filtered Eel River water (black circles). B) Anatoxin-a adsorption by SPATT resin in 125 mL Erlenmeyer flasks filled with 0.2 μm filtered Eel River or Pinto Lake water. No SPATT resin was placed in control flasks (white squares). (TIF) [file pone.0197669.s002.tif]

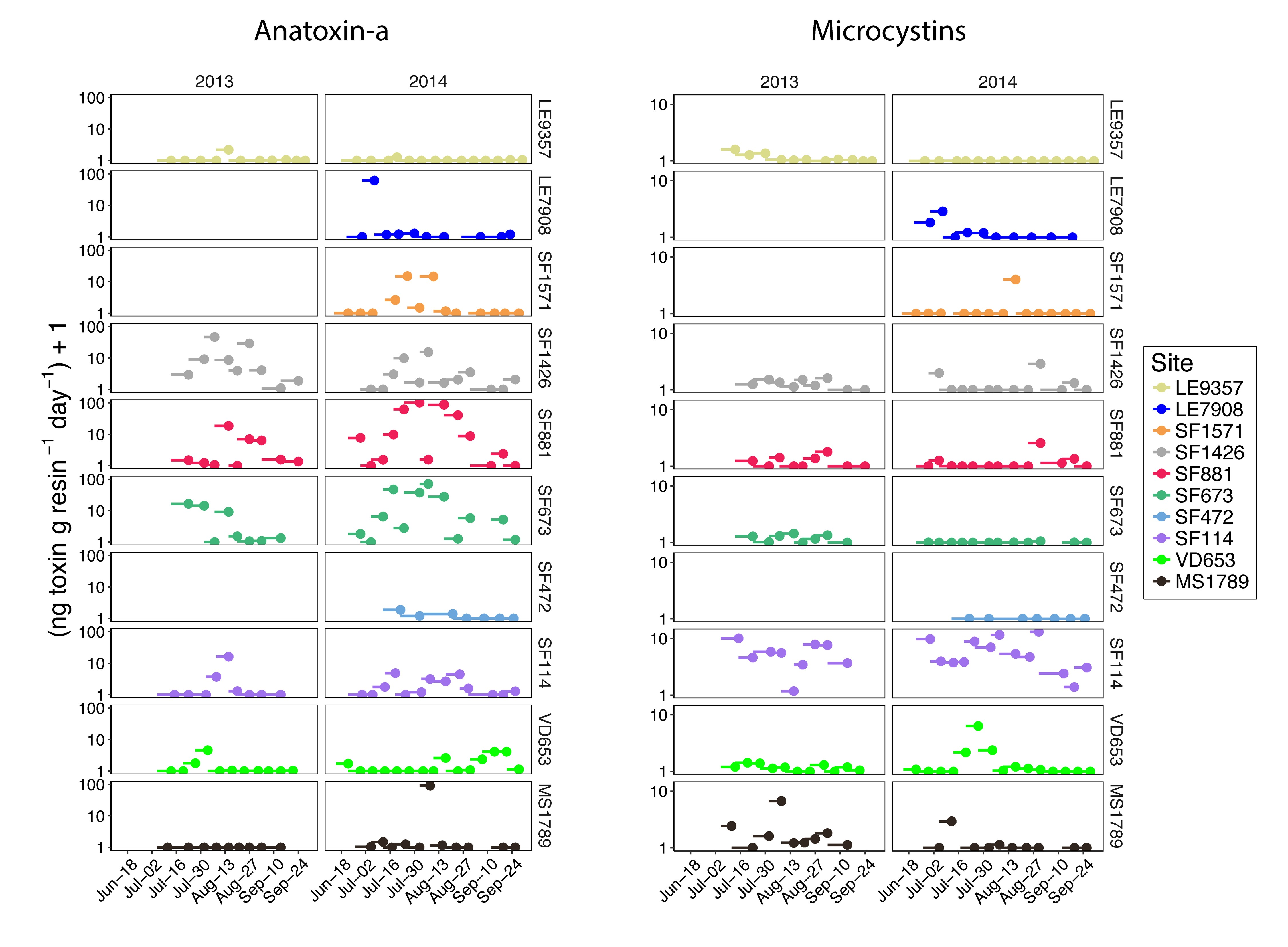

Supplement: S3 Fig — Sites are ordered top to bottom by watershed size. Each horizontal line represents an individual SPATT sampler and the length of the line corresponds to the number of days of deployment. LE7908, SF1571, and SF472 were only established in 2014. (TIF) [file pone.0197669.s003.tif]

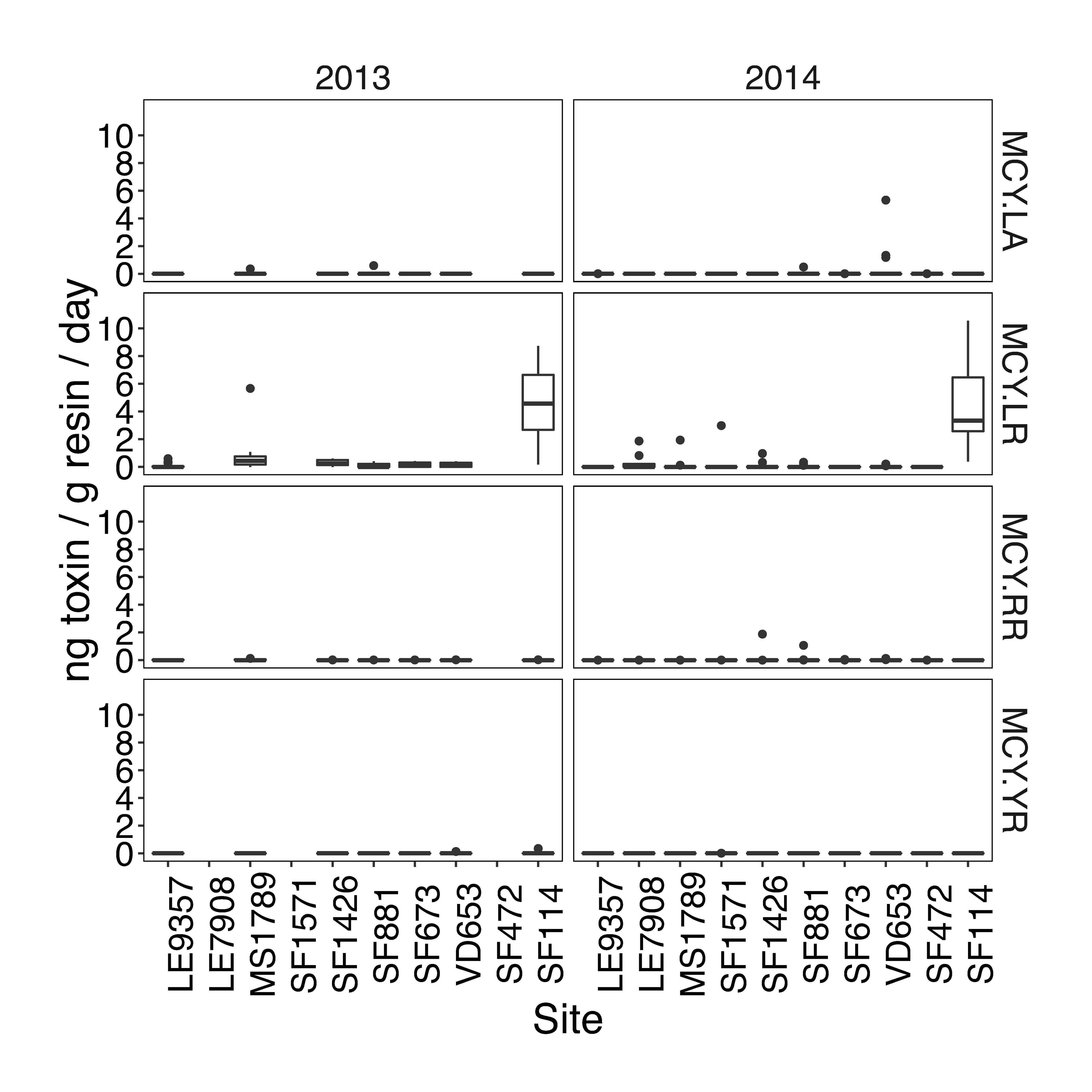

Supplement: S4 Fig — (TIF) [file pone.0197669.s004.tif]

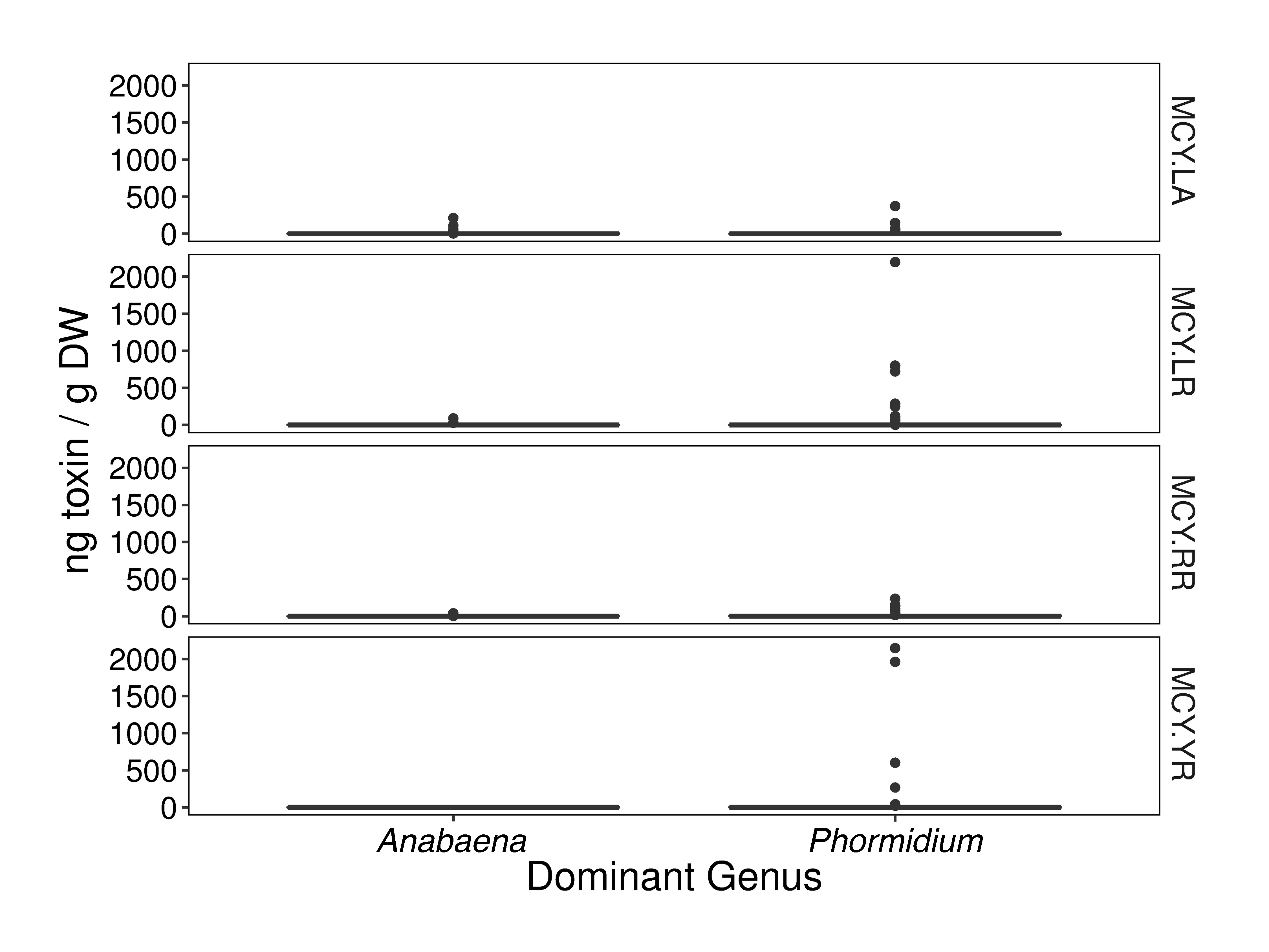

Supplement: S5 Fig — (TIF) [file pone.0197669.s005.tif]

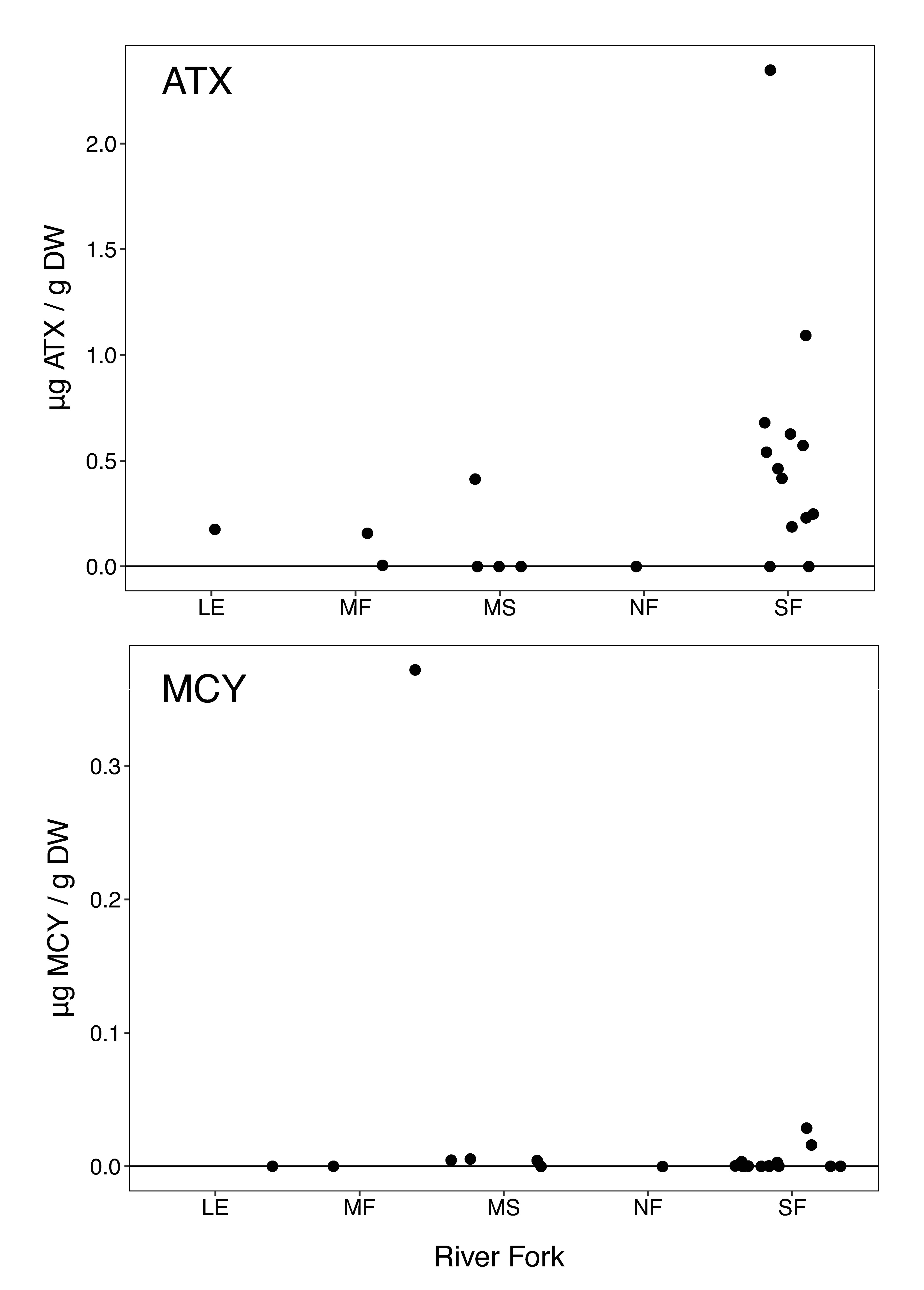

Supplement: S6 Fig — Samples collected in summer 2015 from Lower Eel (LE), Middle Fork (MF), Mainstem (MS), North Fork (NF), and South Fork (SF) Eel. (N = 21, 19 Anabaena samples and 2 Phormidium samples). (TIF) [file pone.0197669.s006.tif]
